# Draft Genome Sequence of Triclosan-Degrading Bacterium *Sphingomonas* sp. Strain YL-JM2C, Isolated from a Wastewater Treatment Plant in China

**DOI:** 10.1128/genomeA.00603-15

**Published:** 2015-06-04

**Authors:** Sikandar I. Mulla, Anyi Hu, Haili Xu, Chang-Ping Yu

**Affiliations:** Key Laboratory of Urban Pollutant Conversion, Institute of Urban Environment, Chinese Academy of Sciences, Xiamen, China

## Abstract

*Sphingomonas* sp. strain YL-JM2C was isolated from a wastewater treatment plant in Xiamen, China, by enrichment on triclosan. The bacterium is of special interest because of its ability to degrade triclosan. Here, we present a draft genome sequence of the microorganism and its functional annotation. To our best knowledge, this is the first report of a draft genome sequence of a triclosan-degrading bacterium

## GENOME ANNOUNCEMENT

Triclosan is an antimicrobial agent used in personal care products, textiles, and plastics (1). Studies demonstrate that triclosan has an acute toxicity to aquatic organisms (2). To date, most of the isolated microorganisms degrading triclosan belong to the sphingomonads (3–5). However, there are no reports available on a complete or draft genome sequence of a triclosan-degrading bacterium. Here, we report a draft genome sequence of the triclosan-degrading *Sphingomonas* sp. strain YL-JM2C, isolated from activated sludge of a wastewater treatment plant in Xiamen, China, through enrichment on triclosan (5 mg liter^-1^). Phylogenetic analysis based on the 16S rRNA sequence showed that the percentage of similarity of YL-JM2C with related species, like *Sphingomonas wittichii* RW1, *Sphingomonas histidinilytica* UM2, and *Sphingomonas haloaromaticamans* A175, ranges from 99.93 to 99.06%.

The YL-JM2C genome was sequenced by an Illumina GAIIx instrument with one paired-end library (300 bp). A total of 594.6 Mb high-quality sequencing data were obtained, having approximately 100-fold coverage. Genome sequences were assembled *in silico* using SOAP*denovo* version 1.05 (6), resulting in 273 contigs (>200 bp), with an *N*_50_ length of 45,887 bp. The gene-caller Glimmer 3.02 was used to predict coding sequences (CDSs), whereas for the identification of rRNA and tRNA, RNAmmer and tRNAscan-SE were used, respectively. For confirmation of the annotation sequence, the genome sequence was further uploaded into the Rapid Annotations using Subsystems Technology (RAST). Comparisons of the functions of predicted protein-coding genes using the NCBI-NR, COG, and KEGG databases were conducted during gene annotation (7).

The draft genome sequence of YL-JM2C consists of 5,923,019 bp, with 67.88% G+C content. From a total of 5,758 genes, 5,711 were protein-coding genes and 47 were RNAs. The majority of genes (69.28%) were assigned a putative function, whereas the remaining genes were either hypothetical proteins or proteins with an unknown function. According to subsystem-based annotation generated by RAST, 44% of protein-coding genes were assigned to 401 metabolic subsystems. In comparison with other *Sphingomonas* strains, YL-JM2C has a bigger genome, with 5,711 protein-coding genes (8).

We performed an average nucleotide identity (ANI) and Genome-to-Genome Distance Calculator (GGDC) using JSpecies version 1.2 (9), and GGDC version 2.0 (10) was used to compare similarities among the genomic sequences of *Sphingomonas* strains YL-JM2C, RW1, DP58, KC8, and PR090111-T3T-6A (8, 11–13). ANI analysis showed YL-JM2C shared a low degree of similarity with other *Sphingomonas* species (<74% ANI), whereas relatively higher values were obtained for RW1 and DP58. The GGDC analysis showed similar results. We also compared orthologs of the genes of five *Sphingomonas* strains. Overall, strains YL-JM2C, RW1, and DP58 shared 3,957 orthologous genes, whereas relatively lower numbers of orthologous genes were identified between YL-JM2C and the other two strains, suggesting that strains YL-JM2C, RW1, and DP58 might belong to the same species. As for the annotation data (for YL-JM2C), the bacterium has 45 monooxygenase and 101 dioxygenase enzymes, which are probably involved in the degradation of diverse pollutants, including triclosan.

### Nucleotide sequence accession numbers.

The draft genome sequence of *Sphingomonas* sp. strain YL-JM2C was deposited in DDBJ/EMBL/GenBank under the accession no. ASTM00000000, and the version described here is the first version, ASTM01000000.
